# Real‐World Safety and Effectiveness of Icatibant in Patients With Hereditary Angioedema (HAE): Post‐Marketing Surveillance in Japan

**DOI:** 10.1111/1346-8138.70447

**Published:** 2026-08-21

**Authors:** Kumiko Hosono, Miyako Hoshino

**Affiliations:** ^1^ Japan Medical Office Takeda Pharmaceutical Company Limited Tokyo Japan

**Keywords:** bradykinin B2 receptor antagonist, hereditary angioedema, icatibant, Japan, on‐demand treatment, real‐world evidence

## Abstract

Hereditary angioedema (HAE) is a rare, autosomal dominant, life‐threatening disease characterized by recurrent attacks of swelling of the face, upper airway, gastrointestinal tract, limbs, and/or genitalia. Genetic mutations of the *SERPING1* gene encoding the C1‐inhibitor (C1‐INH) plasma protein are associated with HAE‐C1INH. HAE is also observed in patients with normal C1‐INH (HAE‐nC1INH). Icatibant is a selective bradykinin B2 receptor antagonist for treatment of acute attacks of HAE. Here we report safety and effectiveness data from a post‐marketing, open‐label, multicenter observational study of icatibant conducted in Japan (ClinicalTrials.gov ID: NCT04057131). Patients with HAE in Japan who initiated icatibant between November 2018 and June 2020 were enrolled; surveillance was completed in April 2023. In the Safety Analysis Set (*n* = 155), most patients were women (*n* = 116, 74.84%) and HAE types included HAE‐C1INH‐Type 1 (*n* = 83, 53.55%), HAE‐C1INH‐Type 2 (*n* = 9, 5.81%), unclassified HAE‐C1INH (*n* = 16, 10.32%), and HAE‐nC1INH (*n* = 22, 14.19%). Overall, 19 patients (12.26%) experienced adverse drug reactions (ADRs), most of which were mild in severity (*n* = 18, 11.61%). The most common ADRs were general disorders and administration site conditions (*n* = 18, 11.61%). No serious ADRs or ADRs of special interest other than hypersensitivity and injection site reaction were reported. In the Effectiveness Analysis Set (*n* = 100), 97 patients (97.00%) received 30 mg of icatibant. Among 417 HAE attacks, 234 were treated with icatibant alone and 48 with icatibant plus other. Among attacks treated with icatibant alone, median (interquartile range) time to first symptom relief was 60.0 (30.0–270.0) min, and median time to complete resolution of symptoms was 710.0 (148.5–1440.0) min. In conclusion, in this study of the real‐world use of icatibant, no new safety concerns were reported. Icatibant was effective in providing early symptom relief for HAE attacks across a broad spectrum of patients, including patients with HAE‐nC1INH, who were not included in previous clinical trials.

## 1 Introduction

Hereditary angioedema (HAE), a rare autosomal dominant disorder, is a serious, debilitating, and life‐threatening condition characterized by unpredictable, intermittent attacks of subcutaneous or submucosal edema of the face, upper airway (larynx), gastrointestinal tract, limbs, and/or genitalia [1, 2]. Mortality related to upper airway attacks is significant (~30%) and is up to 9 times higher in undiagnosed than in diagnosed patients [3]. Triggers of angioedema attacks in patients with HAE can include stress, physical trauma, medical or dental procedures, and estrogen exposure, although most attacks appear to occur spontaneously [2, 4, 5]. The risk of death from asphyxiation and the chronic recurrent and unpredictable nature of HAE attacks considerably affect patient quality of life and mental health and can cause significant disruption to vocational and school achievement [6, 7, 8].

HAE is an orphan disorder that affects all ethnic populations, adults and children, and all sexes [9]. The exact prevalence of HAE is unknown, but current estimates range from around 1 per 50 000 persons (United States and Denmark) to 1 per 1 000 000 persons (Taiwan) [10, 11, 12, 13, 14]. Two types of HAE are associated with mutations in the *SERPING1* gene that encodes the C1‐inhibitor plasma protein (C1‐INH) [2, 15]. HAE‐C1INH‐Type 1 is associated with C1‐INH deficiency, whereas HAE‐C1INH‐Type 2 is associated with C1‐INH dysfunction [2]. HAE is also observed in patients with normal C1‐INH levels and function (HAE‐nC1INH) [1, 16]. Patients with HAE‐nC1INH may carry pathogenic variants in genes encoding coagulation factor XII (*F12*), plasminogen (*PLG*), angiopoietin‐1 (*ANGPT1*), kininogen‐1 (*KNG1*), myoferlin (*MYOF*), heparan sulfate‐glucosamine 3‐O‐sulfotransferase 6 (*HS3ST6*), carboxypeptidase N (*CPN1*), and/or disabled homolog 2‐interacting protein (*DAB2IP*) [16, 17, 18, 19, 20]. In Japan, HAE occurs in an estimated 1 of every 50 000 persons [21], which means there are likely 2000–3000 patients with HAE in Japan [22]. However, previous studies have only included between 46 and 179 patients [22, 23, 24, 25], and it has been estimated that only a total of 450–550 patients have been diagnosed with HAE in Japan, with limited information available about their treatment status [21, 22].

International treatment guidelines for HAE recommend on‐demand treatment with intravenous C1‐INH concentrate, ecallantide, or icatibant [18] to minimize HAE attacks. More recent guidelines also include oral sebetralstat as an on‐demand treatment option [26, 27]. Icatibant (Firazyr, Takeda Pharmaceutical Company, Tokyo, Japan) is a selective bradykinin B2 receptor antagonist for subcutaneous self‐administration for on‐demand treatment [18, 28]. In phase 3 randomized controlled trials and open‐label extensions (FAST‐1/2, FAST‐1 OLE, FAST‐2 OLE, FAST‐3), a single subcutaneous injection of icatibant consistently provided rapid symptom improvement for acute HAE attacks [29, 30, 31, 32, 33]. Evidence for the safety and efficacy of icatibant has also been provided by the phase 3 open‐label study of icatibant for patients with HAE in Japan [34], with clinical trial findings and an overview of treatment of HAE in Japan summarized in a review article [35]. Most of the available information regarding the clinical characteristics and treatment status of Japanese patients with HAE was reported prior to the launch of icatibant, and data following the approval of icatibant remain extremely limited, including the use of icatibant in Japanese patients with HAE.

After the approval of icatibant in Japan on September 21, 2018, post‐marketing surveillance was conducted as required by the Japanese health authority. Under the Japanese post‐marketing surveillance framework, surveillance of all patients with HAE who received icatibant in clinical settings was attempted, with the objective of evaluating safety and effectiveness of icatibant in routine clinical practice. This cohort study was primarily prospective, although some patients were registered retrospectively due to administrative procedures at participating sites. This is the first study in Japan to investigate the real‐world use of icatibant for acute HAE attack management in a cohort of more than 100 patients, which is larger than cohorts reported in previous Japanese studies.

## 2 Materials and Methods

### 2.1 Study Design and Study Population

This was an open‐label, multicenter observational study of the actual clinical use of icatibant for HAE attacks in Japan. The study was conducted from November 2018 to April 2023 (Figure 1). The study procedures complied with the principles of Good Post‐marketing Study Practice (GPSP). In accordance with GPSP, institutional ethics committee approval and written informed consent from patients were not mandatory; however, institutional approval and informed consent were obtained as appropriate in accordance with the requirements of each individual study site. All data were collected anonymously. The study was registered with ClinicalTrials.gov (ID: NCT04057131).

**FIGURE 1:**
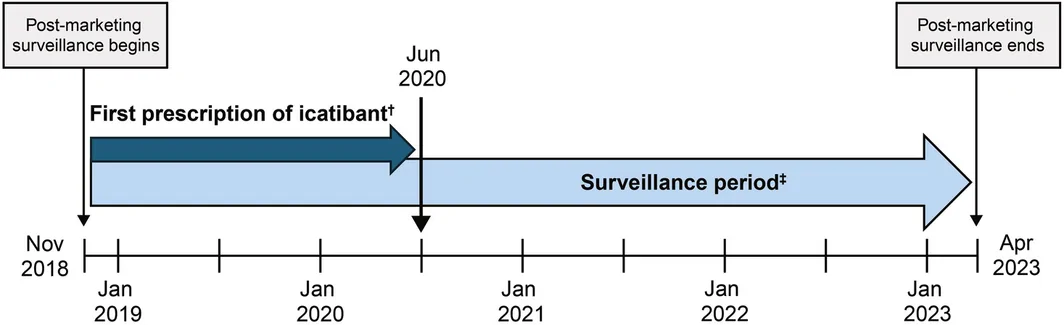
Study timeline. ^†^Patients were eligible for post‐marketing surveillance if they were first prescribed icatibant during this period (November 2018–June 2020). ^‡^Patients were monitored by their treating physician for up to 3 months after use of icatibant; this monitoring occurred any time from the start of post‐marketing surveillance (November 2018) up to the end of post‐marketing surveillance (April 2023).

Patients included in this study had a confirmed diagnosis of HAE, regardless of age or sex, and were administered icatibant for the first time. Patients were eligible for enrollment if they were prescribed icatibant at any time from November 2018 up to June 2020. Patients were primarily enrolled prospectively, but some were enrolled retrospectively (including cases in which prescription and administration of the drug occurred before completion of patient registration). Patients with a history of icatibant administration in a clinical trial prior to approval were excluded.

### 2.2 Treatment and Observation Protocol

Icatibant was administered as a 30‐mg subcutaneous injection, according to the package insert [28]. If the response was insufficient or symptoms recurred, an additional dose of 30 mg could be administered, at least 6 h after the first dose, with a maximum of 3 doses administered per 24 h. Patients were observed by their treating physician for up to 3 months following the first administration of icatibant. This observation period was set based on the results of the Japanese phase 3 clinical trial, in which the median number of months since the last attack prior to the trial was 1.5 months. The reporting period for HAE attack and treatment status was 3 months after the initial attack or until resolution of the fifth HAE attack treated with icatibant for patients with frequent attacks. The observation period for each patient began when icatibant was administered for an attack that occurred after icatibant was prescribed. Study surveillance was completed in April 2023 (Figure 1).

### 2.3 Outcome Measures

#### 2.3.1 Baseline Demographics and Clinical Characteristics

Case report forms (CRFs) were used to record patient characteristics, information about HAE attacks, and information about icatibant administration during the observation period. CRFs were completed by treating physicians based on information provided by patients and routine clinical information available at the study site. Patients were not required to complete a diary for this study. Most sites used electronic CRFs, submitted via an electronic data capture system; at some sites, paper CRFs were completed and either mailed to the sponsor or collected in person by the sponsor's representative. Patient characteristics collected at baseline included patient demographics, HAE diagnosis information (including HAE type and family history), information about concomitant medications, medical history, comorbidities, and history of HAE attacks in the previous 12 months, including number of attacks, attack site(s), and treatments. Source data verification of the CRFs was not performed.

#### 2.3.2 Safety Outcomes

Adverse drug reactions (ADRs) and serious ADRs that occurred during the observation period were coded using the Medical Dictionary for Regulatory Activities, Japanese edition, version 26.0. ADRs of special interest were serious hypersensitivity and severe injection site reaction, aggravation of cardiac function under ischemic conditions due to bradykinin antagonistic action, decreased blood pressure, and immunogenicity. In addition, timing and reasons for any patient discontinuations or withdrawals were recorded.

#### 2.3.3 Effectiveness Outcomes

The administration and response to the use of icatibant in patients who experienced HAE attacks were recorded, including administration status (self‐administered or hospital‐administered), dose administered and number of doses, number and duration of HAE attacks with or without icatibant or other treatment, time to treatment initiation (defined as the time between attack onset and the first injection of treatment), time to first symptom relief, and time to complete resolution of symptoms (defined as the time between the first injection of treatment and the complete resolution of all symptoms).

### 2.4 Statistical Analysis

The planned sample size was 75 patients, based on the number of patients with HAE in Japan, the proportion of patients who received treatment for acute attacks, and the incidence of severe injection site reactions reported in the Japanese phase 3 clinical trial [34]. To collect information from 75 patients, the surveillance period was set to be ~4 years.

Demographic and safety outcomes were analyzed using the Safety Analysis Set, which comprised all registered patients whose CRFs were collected and who received icatibant during the observation period. Safety outcomes are reported using descriptive statistics. The occurrence of ADRs was categorized by treatment type (icatibant alone, icatibant plus other treatment) and by HAE type (HAE‐C1INH or HAE‐nC1INH), as well as by age and pre‐existing conditions.

The Effectiveness Analysis Set consisted of all registered patients whose CRFs were collected, who received icatibant during the observation period, who were not administered icatibant off‐label, and who had evaluable effectiveness information available. In this study, on‐label versus off‐label use was determined in accordance with the indication stated in the Japanese package insert for icatibant, that is, treatment of acute attacks of HAE [28]. Any pediatric administration prior to August 2022 (date of pediatric indication approval) was off‐label, and these patients were excluded. Effectiveness outcomes are reported using descriptive statistics and categorized by HAE type (HAE‐C1INH, HAE‐nC1INH).

Statistical analyses were performed using SAS version 9.4 or later (SAS Institute, Cary, NC, USA) and Microsoft Excel (Microsoft 365) version 16.0 or later (Microsoft Corporation, Redmond, WA, USA).

## 3 Results

### 3.1 Patient Disposition

At the data lock point of June 19, 2023, 179 patients from 93 sites were registered for collection of CRFs (Figure 2). The mean number of registered patients per site was 1.9; the minimum and maximum numbers were 1 and 10, respectively. One patient was excluded because the CRF was not collected. After exclusion of 23 patients who were not treated with icatibant during the observation period, 155 patients were included in the Safety Analysis Set. After exclusion of 42 patients for whom effectiveness was not evaluable, because the time to symptom relief or complete resolution of symptoms was unknown, and 14 patients with off‐label or unknown reasons for use (comprising 11 pediatric patients treated with icatibant before the pediatric indication approval in August 2022 and 3 patients with no gene mutation for HAE and no family history), 100 patients were included in the Effectiveness Analysis Set (1 patient was included in both exclusion groups). The 3 patients with no documented gene mutation and no family history were excluded for potentially off‐label use of icatibant because the possibility that the diagnosis was not HAE could not be ruled out.

**FIGURE 2:**
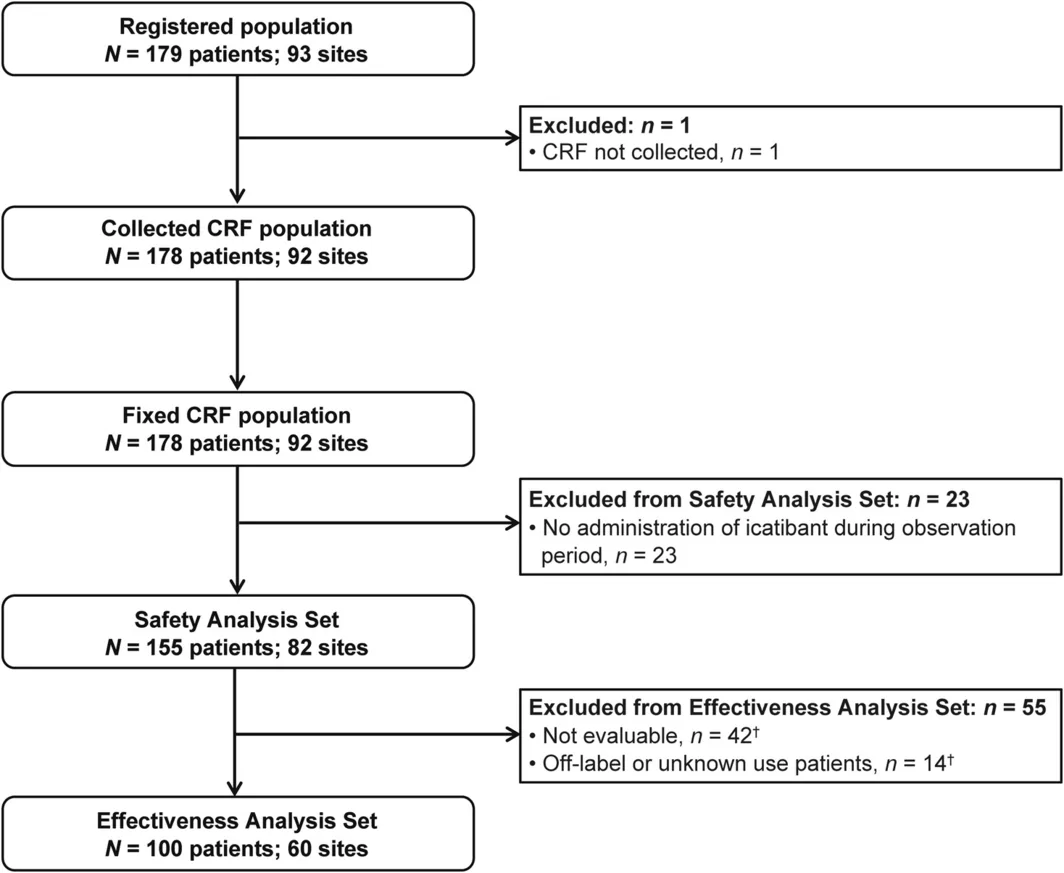
Patient disposition flow chart. ^†^ *n* = 1 patient was included in both exclusion groups (effectiveness not evaluable and off‐label or unknown reason for use). CRF, case report form.

Among the 155 patients included in the Safety Analysis Set, 149 patients (96.13%) continued the study, and 6 patients (3.87%) discontinued. For the 6 patients who discontinued, the reasons for discontinuation were experience of an adverse event in 2 patients (1 case of injection site pain with HAE‐C1INH‐Type 1 and 1 case of drug ineffective with unknown HAE type), no revisit to the survey site in 2 patients, and completion of the survey period in 2 patients.

### 3.2 Demographic and Baseline Clinical Characteristics

Most of the patients in the Safety Analysis Set were female (116 patients, 74.84%) (Table 1). The mean age of patients was 41.6 years, with a minimum age of 7 years and a maximum of 75 years. There were 140 patients (90.32%) who were adults ≥ 18 years of age and 13 patients (8.39%) who were ≥ 65 years of age. Medical and allergy history was known for 31 patients (20.00%) and 34 patients (21.94%), respectively, and 62 patients (40.00%) had a history of comorbidities. Concomitant medications were used by 113 patients (72.90%) and concomitant therapies by 3 patients (1.94%). The most common concomitant medications were tranexamic acid (65 patients, 57.52%) and plasma‐derived C1‐INH (54 patients, 47.79%) (Table S1). Among the 3 patients receiving concomitant therapies, 1 patient each received artificial dialysis, tracheostomy, or hemodialysis maintenance.

**TABLE 1:** Patient demographics (Safety Analysis Set).

Variable	Patients (*N* = 155)
Sex	
Male	38 (24.52)
Female	116 (74.84)
Not recorded	1 (0.65)
Ethnic origin, Japanese	155 (100.00)
Age, years	*N* = 151
Mean	41.6
Minimum	7
Maximum	75
Age categories	
< 18 years	11 (7.10)
≥ 18 years	140 (90.32)
< 65 years	138 (89.03)
≥ 65 years	13 (8.39)
Age not recorded	4 (2.58)
Pregnancies (female only)	*N* = 116
No	112 (96.55)
Yes	4 (3.45)
Lactation (female only)	*N* = 116
No	109 (93.97)
Yes	4 (3.45)
Unknown	3 (2.59)
Weight, kg	
< 50	25 (16.13)
≥ 50 to < 60	34 (21.94)
≥ 60 to < 70	26 (16.77)
≥ 70	16 (10.32)
Not measured	54 (34.84)
BMI, kg/m^2^	
< 18.5	9 (5.81)
≥ 18.5 to < 25.0	61 (39.35)
≥ 25.0	27 (17.42)
Not measured/not specified	58 (37.42)
Medical history recorded	
No	115 (74.19)
Yes	31 (20.00)
Unknown	9 (5.81)
Allergy history	
No	105 (67.74)
Yes	34 (21.94)
Unknown	16 (10.32)
Comorbidities	
No	89 (57.42)
Yes	62 (40.00)
Unknown	4 (2.58)
Concomitant medication	
No	42 (27.10)
Yes	113 (72.90)
Concomitant therapy	
No	152 (98.06)
Yes	3 (1.94)

*Note:* All data are *n* (%) unless otherwise specified.

Abbreviation: BMI, body mass index.

There were 83 patients (53.55%) with HAE‐C1INH‐Type 1, 9 patients (5.81%) with HAE‐C1INH‐Type 2, 16 patients (10.32%) with unclassified HAE‐C1INH, and 22 patients (14.19%) with HAE‐nC1INH; 25 patients (16.13%) were recorded as “unknown” (Table 2). The most common period of time from first HAE attack to first treatment was ≥ 10 years (47 patients, 30.32%), and the most common period of time from HAE diagnosis to first treatment was ≥ 1 to < 5 years (38 patients, 24.52%). Gene mutations were absent in 20 patients (12.90%), present in 33 patients (21.29%), and unknown in 102 patients (65.81%). Family history was absent in 34 patients (21.94%), present in 102 patients (65.81%), and unknown in 19 patients (12.26%).

**TABLE 2:** Patient baseline clinical characteristics (Safety Analysis Set).

Variable	Patients (*N* = 155)
HAE type	
HAE‐C1INH	108 (69.68)
HAE‐C1INH‐Type 1	83 (53.55)
HAE‐C1INH‐Type 2	9 (5.81)
Unclassified	16 (10.32)
HAE‐nC1INH	22 (14.19)
Unknown	25 (16.13)
Time from first attack to first treatment, years	
< 1	1 (0.65)
≥ 1 to < 5	13 (8.39)
≥ 5 to < 10	14 (9.03)
≥ 10	47 (30.32)
Unknown/not specified	80 (51.61)
Time from HAE diagnosis to first treatment, years	
< 1	15 (9.68)
≥ 1 to < 5	38 (24.52)
≥ 5 to < 10	25 (16.13)
≥ 10	25 (16.13)
Unknown/not specified	52 (33.55)
Gene mutation	
No	20 (12.90)
Yes	33 (21.29)
Unknown	102 (65.81)
Family history	
No	34 (21.94)
Yes	102 (65.81)
Father^a^	24 (23.53)
Mother^a^	48 (47.06)
Brother/sister^a^	39 (38.24)
Grandparents^a^	21 (20.59)
Others^a^	36 (35.29)
Unknown	19 (12.26)
History of HAE attacks in the 12 months before icatibant administration	
Number of HAE attacks	
0	7 (4.52)
≥ 1 to < 10	59 (38.06)
≥ 10 to < 25	34 (21.94)
≥ 25 to < 50	9 (5.81)
≥ 50	12 (7.74)
Unknown	34 (21.94)
Number of treatments required^b^	
< 10	73 (49.32)
≥ 10 to < 25	17 (11.49)
≥ 25 to < 50	8 (5.41)
≥ 50	8 (5.41)
Unknown	42 (28.38)
Location of HAE attack^b^, ^c^	
Laryngeal	40 (27.03)
Cutaneous	102 (68.92)
Abdominal	109 (73.65)
Other	14 (9.46)
Treatments administered for HAE attacks^b^, ^d^	
Plasma‐derived C1‐INH	116 (78.38)
Tranexamic acid	60 (40.54)
Endotracheal intubation/tracheostomy	2 (1.35)
Other	15 (10.14)
Time between most recent HAE attack and start of icatibant administration^b^	
< 1 week	12 (8.11)
≥ 1 week to < 1 month	24 (16.22)
≥ 1 month to < 3 months	38 (25.68)
≥ 3 months to < 6 months	13 (8.78)
≥ 6 months to < 1 year	12 (8.11)
≥ 1 year	1 (0.68)
Unknown	48 (32.43)
Training for self‐administration of icatibant	
Not performed	14 (9.03)
Performed	141 (90.97)
Training target: patient^e^	140 (99.29)
Training target: other than patient^e^	1 (0.71)

*Note:* All data are *n* (%) unless otherwise specified.

Abbreviations: C1‐INH, C1‐inhibitor; HAE, hereditary angioedema; HAE‐C1INH, HAE with C1‐INH deficiency (Type 1) or dysfunction (Type 2); HAE‐nC1INH, HAE with normal C1‐INH.

^a^ Denominator for the percentage is all patients with a family history (*n* = 102).

^b^ Denominator for the percentage is all patients who experienced an attack (*n* = 148).

^c^ Individual patients could have multiple sites affected by HAE attacks.

^d^ Individual patients could receive multiple treatments.

^e^ Denominator for the percentage is all patients who received training for self‐administration (*n* = 141).

The most common number of HAE attacks in the 12 months before icatibant administration was < 10 (59 patients, 38.06%), but it was also common to have had ≥ 10 to < 25 attacks (34 patients, 21.94%) (Table 2). For HAE attacks in the 12 months prior to icatibant administration, the most common number of treatments was < 10 (73 patients, 49.32%). Individual patients could have multiple sites affected by HAE attacks, and both abdominal (109 patients, 73.65%) and cutaneous (102 patients, 68.92%) sites were common in the 12 months prior to treatment with icatibant. Among patients who had an HAE attack (*n* = 148), the most commonly administered treatment type was plasma‐derived C1‐INH (116 patients, 78.38%), followed by tranexamic acid (60 patients, 40.54%), other treatment type (15 patients, 10.14%), and intratracheal inoculation (2 patients, 1.35%); individual patients could have multiple treatments administered.

### 3.3 Safety Outcomes

Overall, there were 19/155 patients (12.26%) who experienced ADRs (Table 3). Most of the ADRs recorded were administration site reactions and included injection site pain, erythema, reaction, urticaria, swelling, and warmth. Other ADRs included headache, malaise, and fibrin D dimer increased. The event outcome was recovered for all ADRs except 1 case each of injection site pain and fibrin D dimer increased that were not recovered and 1 case each of injection site urticaria and malaise that were recovering. No serious ADRs were observed. Among the ADRs of special interest, although hypersensitivity/administration site reactions were reported in 17/155 patients (10.97%), none of these were serious or severe, and there were no cases observed of aggravation of cardiac function under ischemic conditions due to bradykinin antagonistic action, blood pressure decreased, or immunogenicity.

**TABLE 3:** ADRs and infections (Safety Analysis Set).

	Overall (*N* = 155)	HAE type			Severity (*N* = 155)			Treatment	
		HAE‐C1INH (*N* = 108)	HAE‐nC1INH (*N* = 22)	Unknown (*N* = 25)	Mild	Moderate	Severe	Icatibant alone (*N* = 94)	Icatibant plus other treatment (*N* = 61)
Patients with ≥ 1 ADR	19 (12.26)	15 (13.89)	3 (13.64)	1 (4.00)	18 (11.61)	2 (1.29)	0 (0.00)	11 (11.70)	8 (13.11)
SOC^a^									
PT^a^									
Nervous system disorders	1 (0.65)	1 (0.93)	0 (0.00)	0 (0.00)	1 (0.65)	0 (0.00)	0 (0.00)	0 (0.00)	1 (1.64)
Headache	1 (0.65)	1 (0.93)	0 (0.00)	0 (0.00)	1 (0.65)	0 (0.00)	0 (0.00)	0 (0.00)	1 (1.64)
General disorders and administration site conditions	18 (11.61)	14 (12.96)	3 (13.64)	1 (4.00)	16 (10.32)	2 (1.29)	0 (0.00)	10 (10.64)	8 (13.11)
Injection site erythema	5 (3.23)	4 (3.70)	0 (0.00)	1 (4.00)	5 (3.23)	0 (0.00)	0 (0.00)	3 (3.19)	2 (3.28)
Injection site pain	7 (4.52)	7 (6.48)	0 (0.00)	0 (0.00)	6 (3.87)	1 (0.65)	0 (0.00)	3 (3.19)	4 (6.56)
Injection site reaction	4 (2.58)	2 (1.85)	2 (9.09)	0 (0.00)	4 (2.58)	0 (0.00)	0 (0.00)	2 (2.13)	2 (3.28)
Injection site urticaria	2 (1.29)	1 (0.93)	1 (4.55)	0 (0.00)	2 (1.29)	0 (0.00)	0 (0.00)	2 (2.13)	0 (0.00)
Injection site warmth	1 (0.65)	1 (0.93)	0 (0.00)	0 (0.00)	1 (0.65)	0 (0.00)	0 (0.00)	0 (0.00)	1 (1.64)
Malaise	1 (0.65)	1 (0.93)	0 (0.00)	0 (0.00)	0 (0.00)	1 (0.65)	0 (0.00)	0 (0.00)	1 (1.64)
Injection site swelling	2 (1.29)	2 (1.85)	0 (0.00)	0 (0.00)	1 (0.65)	0 (0.00)	0 (0.00)	0 (0.00)	2 (3.28)
Investigations	1 (0.65)	1 (0.93)	0 (0.00)	0 (0.00)	1 (0.65)	0 (0.00)	0 (0.00)	1 (1.06)	0 (0.00)
Fibrin D dimer increased	1 (0.65)	1 (0.93)	0 (0.00)	0 (0.00)	1 (0.65)	0 (0.00)	0 (0.00)	1 (1.06)	0 (0.00)

*Note:* All data are *n* (%).

Abbreviations: ADR, adverse drug reaction; C1‐INH, C1‐inhibitor; HAE, hereditary angioedema; HAE‐C1INH, HAE with C1‐INH deficiency (Type 1) or dysfunction (Type 2); HAE‐nC1INH, HAE with normal C1‐INH; PT, preferred term; SOC, system organ class.

^a^ SOCs and PTs from Medical Dictionary for Regulatory Activities, Japanese edition, version 26.0.

When ADRs were examined by HAE type, 15/108 patients (13.89%) with HAE‐C1INH, 3/22 patients (13.64%) with HAE‐nC1INH, and 1/25 patients (4.00%) with unknown HAE type experienced ADRs (Table 3). No patients experienced severe ADRs, 2 patients (1.29%) experienced moderate severity ADRs, and 18 patients (11.61%) experienced mild ADRs. The moderate severity ADRs observed were injection site pain in 1 patient (0.65%) and malaise in 1 patient (0.65%). ADRs were observed in 11/94 patients (11.70%) treated with icatibant alone and 8/61 patients (13.11%) treated with icatibant plus other treatment. The number of patients who developed each ADR was similar in each treatment group, and no particular trend was observed relating to treatment.

No significant difference was observed in the incidence rate of ADRs by age group (i.e., age group in 10‐year increments, age group by pediatric category [< 18 years vs. ≥ 18 years], age group by geriatric category [< 65 years vs. ≥ 65 years]; data not shown). The incidence of ADRs in children, elderly adults, and pregnant/nursing women was 27.27% (3/11 patients), 15.38% (2/13 patients), and 0.00% (0/7 patients), respectively.

Among 7 patients with HAE in their 30–40 s, 4 cases of maternal icatibant exposure during pregnancy and 4 cases of maternal icatibant exposure during breastfeeding were confirmed. The HAE types for patients with maternal exposure were either HAE‐C1INH‐Type 1 or unknown. No adverse events were reported during the study for these patients. Furthermore, in at least 1 of the 4 cases of maternal exposure during pregnancy, the report indicated that no abnormalities (adverse events) occurred in the mother or the fetus.

### 3.4 Effectiveness Outcomes

#### 3.4.1 Treatment Details

The total number of acute attacks during the observation period was 417 episodes, of which 377 received treatment (Figure 3a). Among patients with HAE‐C1INH, 323 acute attacks occurred, of which 300 received treatment (Figure 3b). Among patients with HAE‐nC1INH, 37 acute attacks occurred, of which 32 received treatment (Figure 3c). Most treated attacks were treated with icatibant alone (234/377 attacks overall, 182/300 attacks among patients with HAE‐C1INH, and 18/32 attacks among patients with HAE‐nC1INH; Figure 3).

**FIGURE 3:**
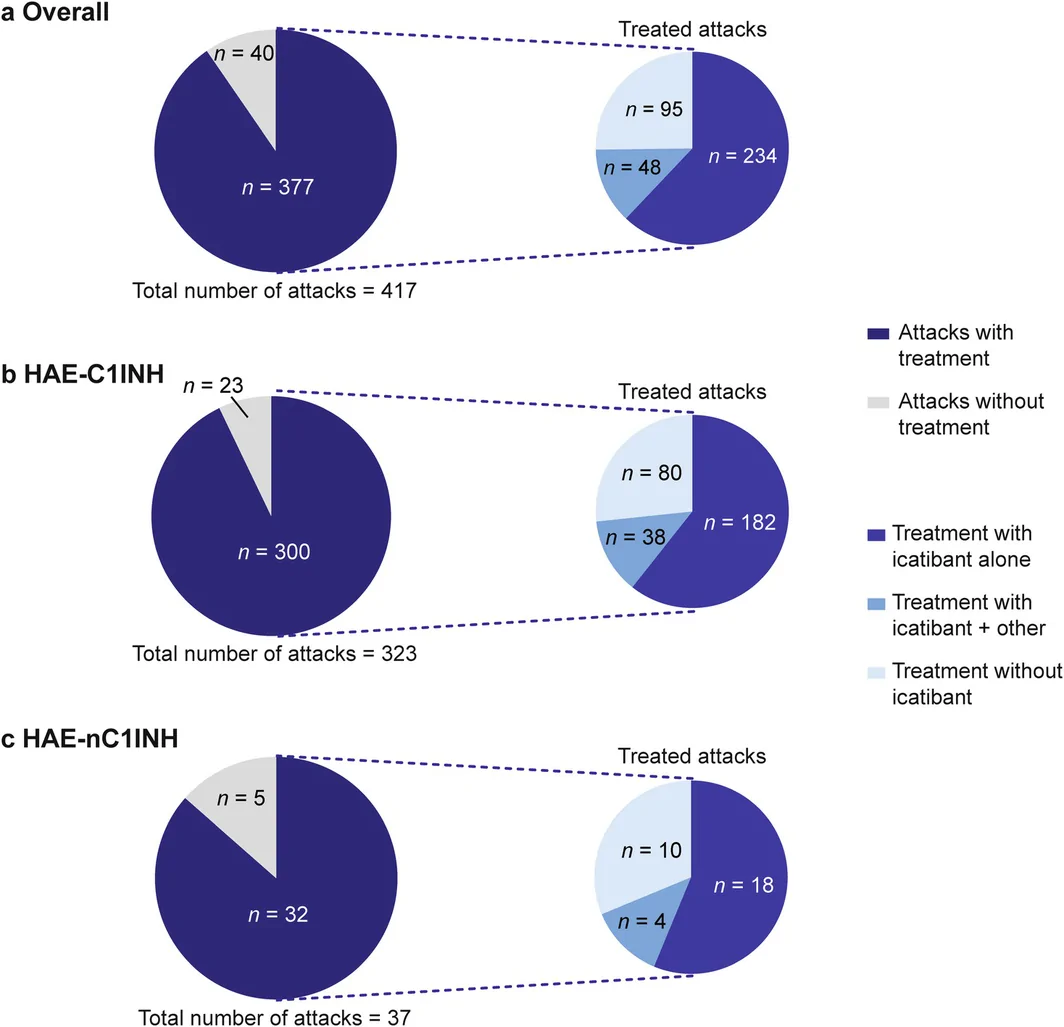
Treatment for HAE attacks during the observation period (Effectiveness Analysis Set). (a) All patients; (b) HAE‐C1INH; (c) HAE‐nC1INH. C1‐INH, C1‐inhibitor; HAE, hereditary angioedema; HAE‐C1INH, HAE with C1‐INH deficiency (Type 1) or dysfunction (Type 2); HAE‐nC1INH, HAE with normal C1‐INH.

During the observation period, 97 patients (97.00%) in the Effectiveness Analysis Set received a 30‐mg dose of icatibant, 2 patients (2.00%) received 60 mg, and the dose was changed for 1 patient (1.00%) depending on administration time (Table 4). The 1 patient who changed from 60 to 30 mg and 1 of the patients who received 60 mg were patients with HAE‐C1INH. The other patient who received 60 mg was a patient with HAE‐nC1INH.

**TABLE 4:** HAE attacks during the observation period (Effectiveness Analysis Set).^a^

Variable	All patients (*N* = 100)	HAE‐C1INH (*N* = 72)	HAE‐nC1INH (*N* = 10)
Dose of icatibant administered			
30 mg	97 (97.00)	70 (97.22)	9 (90.00)
Changed from 60 to 30 mg^b^	1 (1.00)	1 (1.39)	0 (0.00)
60 mg	2 (2.00)	1 (1.39)	1 (10.00)
Number of attacks per patient			
1	33 (33.00)	25 (34.72)	3 (30.00)
2	21 (21.00)	15 (20.83)	2 (20.00)
3	13 (13.00)	10 (13.89)	1 (10.00)
4	7 (7.00)	5 (6.94)	0 (0.00)
≥ 5	26 (26.00)	17 (23.61)	4 (40.00)
Number of treatment times per attack^c^			
1	337 (89.39)	267 (89.00)	29 (90.63)
2	29 (7.69)	25 (8.33)	1 (3.13)
3	8 (2.12)	6 (2.00)	1 (3.13)
4	3 (0.80)	2 (0.67)	1 (3.13)
≥ 5	0 (0.00)	0 (0.00)	0 (0.00)
Number of treatment times per attack among attacks treated with icatibant alone^d^			
1	228 (97.44)	176 (96.70)	18 (100.00)
2	3 (1.28)	3 (1.65)	0 (0.00)
3	3 (1.28)	3 (1.65)	0 (0.00)
4	0 (0.00)	0 (0.00)	0 (0.00)
≥ 5	0 (0.00)	0 (0.00)	0 (0.00)
Number of treatment times per attack among attacks treated with icatibant plus other treatment(s)^e^			
1	15 (31.25)	12 (31.58)	1 (25.00)
2	25 (52.08)	21 (55.26)	1 (25.00)
3	5 (10.42)	3 (7.89)	1 (25.00)
4	3 (6.25)	2 (5.26)	1 (25.00)
≥ 5	0 (0.00)	0 (0.00)	0 (0.00)
Affected site(s)^c^, ^f^			
Laryngeal	27 (7.16)	18 (6.00)	8 (25.00)
Cutaneous	198 (52.52)	152 (50.67)	17 (53.13)
Abdominal	171 (45.36)	138 (46.00)	20 (62.50)
Others	58 (15.38)	44 (14.67)	6 (18.75)
Severity of attack^c^			
Mild	128 (33.95)	100 (33.33)	8 (25.00)
Moderate	224 (59.42)	182 (60.67)	19 (59.38)
Severe	22 (5.84)	17 (5.67)	3 (9.38)
Very severe	3 (0.80)	1 (0.33)	2 (6.25)
Treatment method^c^			
Outpatient	149 (39.52)	110 (36.67)	17 (53.13)
Inpatient	13 (3.45)	8 (2.67)	3 (9.38)
Self‐administration	237 (62.86)	201 (67.00)	13 (40.63)

*Note:* All data are *n* (%).

Abbreviations: C1‐INH, C1‐inhibitor; HAE, hereditary angioedema; HAE‐C1INH, HAE with C1‐INH deficiency (Type 1) or dysfunction (Type 2); HAE‐nC1INH, HAE with normal C1‐INH.

^a^ The total number of attacks was 417. The number of attacks with treatment was 377, including 234 attacks treated with icatibant alone, 48 attacks treated with icatibant plus other treatment(s), and 95 attacks treated without icatibant. There were 40 attacks without any treatment.

^b^ Dose different for different attacks.

^c^ Denominator for percentages is the total number of HAE attacks with treatment (overall: *n* = 377; HAE‐C1INH: *n* = 300; HAE‐nC1INH: *n* = 32).

^d^ Denominator for percentages is the total number of HAE attacks treated with icatibant alone (overall: *n* = 234; HAE‐C1INH: *n* = 182; HAE‐nC1INH: *n* = 18).

^e^ Denominator for percentages is the total number of HAE attacks treated with icatibant plus other treatment(s) (overall: *n* = 48; HAE‐C1INH: *n* = 38; HAE‐nC1INH: *n* = 4).

^f^ Some HAE attacks affected multiple sites.

Overall, the most common number of attacks per patient during the observation period was 1 (all patients: 33 patients, 33.00%; HAE‐C1INH: 25 patients, 34.72%; HAE‐nC1INH: 3 patients, 30.00%); it was also relatively common to have ≥ 5 attacks (all patients: 26 patients, 26.00%; HAE‐C1INH: 17 patients, 23.61%; HAE‐nC1INH: 4 patients, 40.00%) (Table 4). Most attacks required only a single treatment (administration) per attack (all patients: 337/377 episodes, 89.39%; HAE‐C1INH: 267/300 episodes, 89.00%; HAE‐nC1INH: 29/32 episodes, 90.63%). Among attacks treated with icatibant alone, most attacks required 1 treatment with icatibant (all patients: 228/234 episodes, 97.44%; HAE‐C1INH: 176/182 episodes, 96.70%; HAE‐nC1INH: 18/18 episodes, 100.00%). Among attacks treated with icatibant plus other treatment(s), there was more variation in the number of treatments required per attack. There was also variation in the order of treatment; for example, some patients received icatibant first, some received plasma‐derived C1‐INH first, and some received icatibant and other treatment(s) at the same time (data not shown). For patients with HAE‐nC1INH, there were 4 attacks in 2/10 patients treated with icatibant plus other treatment(s).

Cutaneous HAE attacks occurred in 198/377 episodes (52.52%) overall, in 152/300 episodes (50.67%) among patients with HAE‐C1INH, and in 17/32 episodes (53.13%) among patients with HAE‐nC1INH (Table 4). Abdominal HAE attacks occurred in 171/377 episodes (45.36%) overall, in 138/300 episodes (46.00%) among patients with HAE‐C1INH, and in 20/32 episodes (62.50%) among patients with HAE‐nC1INH. Laryngeal attacks were the least common site overall (27/377 episodes, 7.16%) and among patients with HAE‐C1INH (18/300 episodes, 6.00%) but the third most common site among patients with HAE‐nC1INH (8/32 episodes, 25.00%).

More than half of HAE attacks during the observation period were moderate in severity (all patients: 224/377 episodes, 59.42%; HAE‐C1INH: 182/300 episodes, 60.67%; HAE‐nC1INH: 19/32 episodes, 59.38%). Severe and very severe attacks were reported for 22/377 episodes overall (5.84%; HAE‐C1INH: 17/300 episodes, 5.67%; HAE‐nC1INH: 3/32 episodes, 9.38%) and 3/377 episodes overall (0.80%; HAE‐C1INH: 1/300 episodes, 0.33%; HAE‐nC1INH: 2/32 episodes, 6.25%), respectively.

The majority of HAE attacks overall and among patients with HAE‐C1INH were treated by self‐administration (all patients: 237/377 episodes, 62.86%; HAE‐C1INH: 201/300 episodes, 67.00%) (Table 4). However, among patients with HAE‐nC1INH, outpatient treatment was the most common treatment method (17/32 episodes, 53.13%).

#### 3.4.2 Treatment Effectiveness

The median time from attack onset to treatment initiation was 120.0 min (interquartile range [IQR]: 30.0–360.0) for all patients, 120.0 (30.0–300.0) min for patients with HAE‐C1INH, and 960.0 (35.0–2215.0) min for patients with HAE‐nC1INH (Table 5). For attacks treated with icatibant alone, median (IQR) time from treatment initiation to first symptom relief was 60.0 (30.0–270.0) min for all patients, 60.0 (30.0–420.0) min for patients with HAE‐C1INH, and 77.5 (30.0–145.0) min for patients with HAE‐nC1INH. For attacks treated regardless of drug, median (IQR) time to first symptom relief was 60.0 (30.0–450.0) min for all patients, 60.0 (30.0–595.0) min for patients with HAE‐C1INH, and 95.0 (30.0–145.0) min for patients with HAE‐nC1INH.

**TABLE 5:** Summary of treatment statistics for HAE attacks during the observation period (Effectiveness Analysis Set).

	All patients (*N* = 100)	HAE‐C1INH (*N* = 72)	HAE‐nC1INH (*N* = 10)
Time from attack onset to treatment initiation, min			
Number of attacks aggregated	163	122	11
Median (IQR)	120.0 (30.0–360.0)	120.0 (30.0–300.0)	960.0 (35.0–2215.0)
Time from treatment initiation to first symptom relief, min			
Overall			
Number of attacks aggregated	128	97	9
Median (IQR)	60.0 (30.0–450.0)	60.0 (30.0–595.0)	95.0 (30.0–145.0)
Icatibant alone			
Number of attacks aggregated	86	66	6
Median (IQR)	60.0 (30.0–270.0)	60.0 (30.0–420.0)	77.5 (30.0–145.0)
Time from treatment initiation to complete resolution of symptoms, min			
Overall			
Number of attacks aggregated	141	109	9
Median (IQR)	780.0 (210.0–1800.0)	930.0 (295.0–2000.0)	1440.0 (130.0–2880.0)
Icatibant alone			
Number of attacks aggregated	96	74	6
Median (IQR)	710.0 (148.5–1440.0)	780.0 (210.0–1620.0)	1440.0 (130.0–2850.0)

Abbreviations: C1‐INH, C1‐inhibitor; HAE, hereditary angioedema; HAE‐C1INH, HAE with C1‐INH deficiency (Type 1) or dysfunction (Type 2); HAE‐nC1INH, HAE with normal C1‐INH; IQR, interquartile range; min, minutes.

For attacks treated with icatibant alone, median time from treatment initiation to complete resolution of symptoms was 710.0 (148.5–1440.0) min for all patients, 780.0 (210.0–1620.0) min for patients with HAE‐C1INH, and 1440.0 (130.0–2850.0) min for patients with HAE‐nC1INH. For attacks treated regardless of drug, median (IQR) time from treatment initiation to complete resolution of symptoms was 780.0 (210.0–1800.0) min for all patients, 930.0 (295.0–2000.0) min in patients with HAE‐C1INH, and 1440.0 (130.0–2880.0) min for patients with HAE‐nC1INH.

Overall, for ~70% of attacks, both the time from attack onset to treatment initiation and the time from treatment initiation to first symptom relief were < 4 h each (Figure 4). Among attacks treated with icatibant alone, the time from treatment initiation to first symptom relief was ≤ 4 h for ~75% of attacks. The time from treatment initiation to complete resolution of symptoms was < 24 h for ~60% of both attacks overall and attacks treated with icatibant alone. Among patients with HAE‐C1INH, the time from attack onset to treatment initiation was < 4 h for ~70% of attacks, the time from treatment initiation to first symptom relief with icatibant alone was < 2 h for ~70% of attacks, and the time to complete resolution of symptoms with icatibant alone was < 24 h for ~60% of attacks (Figure 5). Among patients with HAE‐nC1INH, ~35% of attacks were treated within 4 h, the time from treatment initiation to first symptom relief (icatibant alone) was < 4 h for > 80% of attacks, and ~33% of attacks were completely resolved with icatibant alone within 24 h. For all patients, when treatment and resolution times were stratified by site of attack, the time from attack onset to treatment initiation was < 4 h for ~45% of laryngeal attacks and ~70% of cutaneous and abdominal attacks (Figure 6). The time from treatment initiation to first symptom relief with icatibant alone was < 4 h for ~85% of laryngeal, ~60% of cutaneous, and ~75% of abdominal attacks. Around 55% of laryngeal and cutaneous attacks and ~50% of abdominal attacks were completely resolved with icatibant alone within 24 h.

**FIGURE 4:**
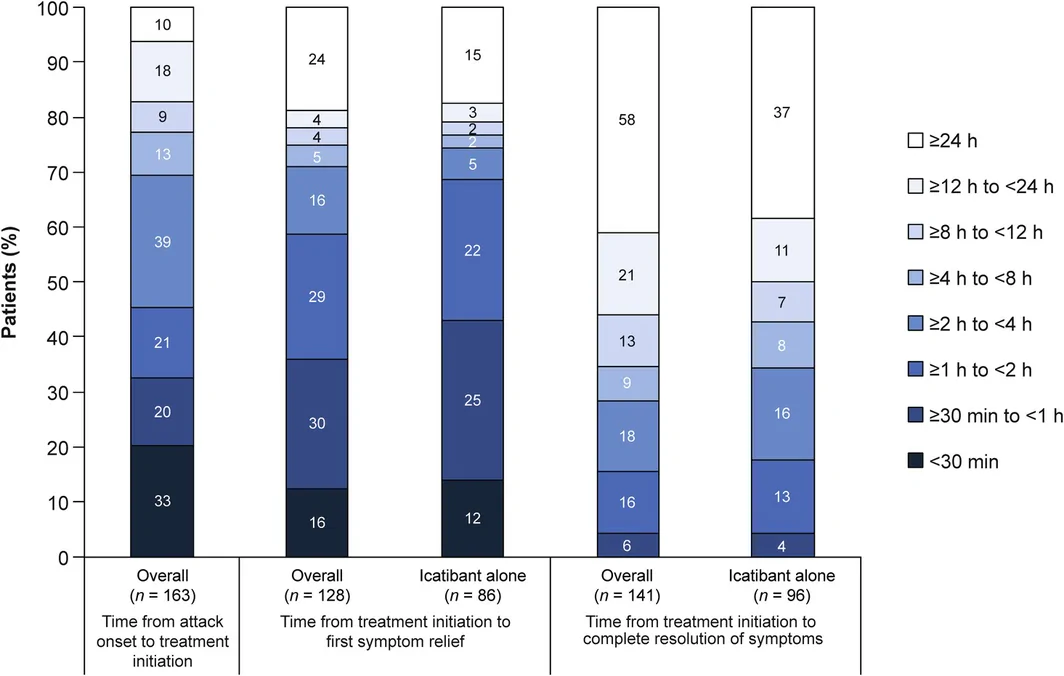
Treatment status of acute attacks for all patients (Effectiveness Analysis Set). Data labels inside each column are the number of attacks in each time window; *n* values at the bottom of each column are the total number of attacks in each category. h, hour; min, minutes.

**FIGURE 5:**
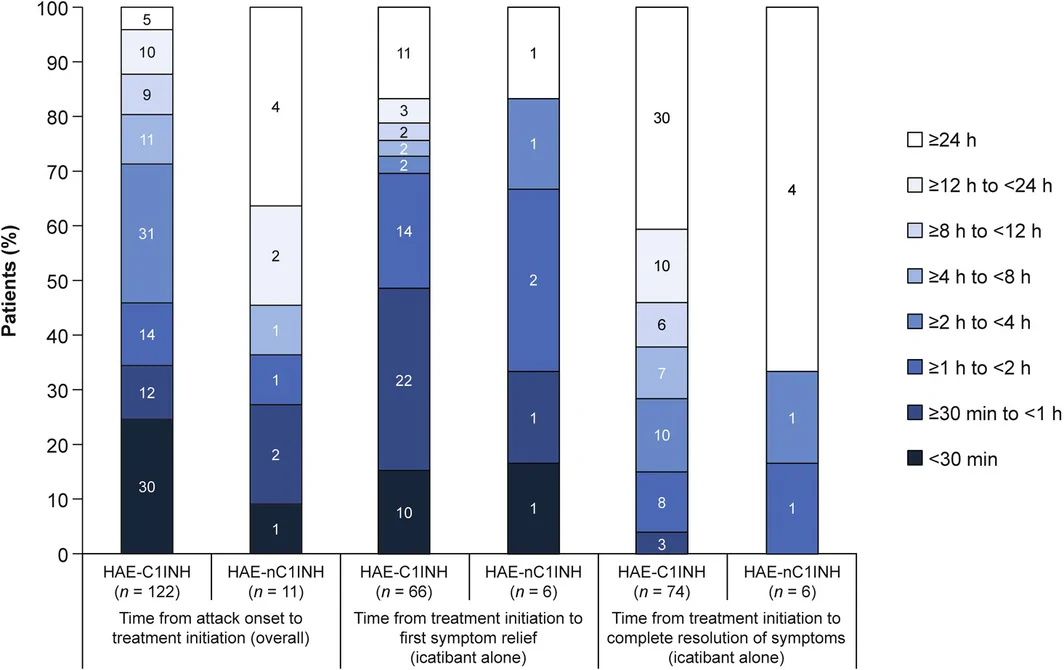
Treatment status of acute attacks, stratified by HAE type (Effectiveness Analysis Set). Data labels inside each column are the number of attacks in each time window; *n* values at the bottom of each column are the total number of attacks in each category. C1‐INH, C1‐inhibitor; h, hour; HAE, hereditary angioedema; HAE‐C1INH, HAE with C1‐INH deficiency (Type 1) or dysfunction (Type 2); HAE‐nC1INH, HAE with normal C1‐INH; min, minutes.

**FIGURE 6:**
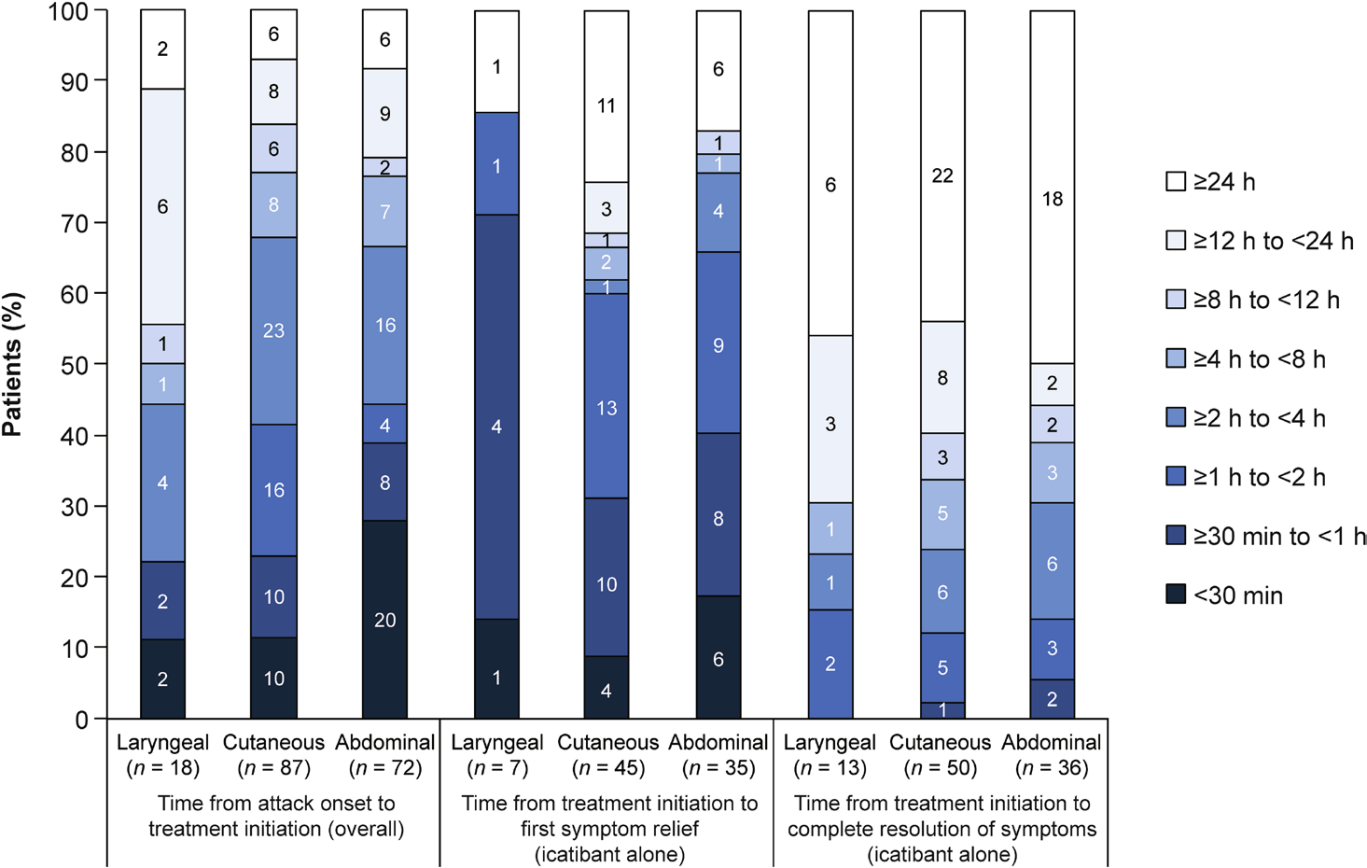
Treatment status of acute attacks, stratified by HAE attack site (Effectiveness Analysis Set). Data labels inside each column are the number of attacks in each time window; *n* values at the bottom of each column are the total number of attacks in each category. h, hour; min, minutes.

## 4 Discussion

This study is the first post‐marketing surveillance of icatibant used for HAE attacks in Japan. The analysis was based on real‐world clinical data and included patients with HAE‐nC1INH and those taking concomitant medications who were not included in previous clinical trials.

During the study, no serious ADRs were reported. All ADRs observed were mild in severity except for injection site pain and malaise, which were recorded at moderate severity in 1 patient each. Most of the ADRs observed were administration site reactions. The most frequent administration site reaction was pain, followed by erythema, unspecified reaction, urticaria, warmth, and swelling. This is consistent with the results of the Japanese phase 3 clinical trial, in which the most frequent administration site reactions (recorded as treatment‐emergent adverse events) were erythema, swelling, and warmth [34]. In the global FAST clinical trials, almost all patients treated with icatibant experienced administration site reactions (recorded separately from adverse events), the most common being erythema and swelling [29, 31, 33]. In the current study, ADRs that were not expected based on the precautions for use for icatibant were malaise (moderate/resolving) and increased fibrin D dimer (mild/not resolved) in 1 patient each. As fibrin D dimer is often elevated during HAE attacks [36], an increase in fibrin D dimer may be due to the underlying disease. The occurrence of ADRs in this study indicates that there were no serious safety issues in the real clinical practice use of icatibant. It was difficult to determine any trends in ADRs reported in relation to patient demographics or clinical characteristics, including type of HAE, because of the small number of patients and/or ADRs in each subgroup.

This study also provides a snapshot of people with HAE in Japan. All participants were Japanese, and the proportion of patients receiving icatibant who were female was 74.84%. In addition to patients with HAE‐C1INH (*n* = 108), the cohort also included patients with HAE‐nC1INH (*n* = 22). The study population covered a wide range of ages, including children aged ≥ 7 years and elderly patients aged ≥ 65 years. Concomitant medications were used by 72.90% of patients, and administration of icatibant during pregnancy and/or lactation was observed in 7 patients. Thus, data were collected from a broad spectrum of patients who were not included in clinical trials.

Of note, this study analyzed data from patients who were prescribed icatibant up to June 2020, during which no new prophylactic agents for acute attacks were available in Japan. Concomitant medications for HAE administered in this study were tranexamic acid (65 patients), plasma‐derived C1‐INH (54 patients), and danazol (7 patients). In Japan, berotralstat, lanadelumab, plasma‐derived C1‐INH, and garadacimab have since been approved for prophylaxis to prevent HAE attacks (approved 2021–2025) [37]; tranexamic acid and danazol are not approved for HAE prophylaxis.

Regarding the characteristics of HAE attacks prior to icatibant administration, 38.06% of patients had experienced ≥ 1 to < 10 attacks in the past 12 months; 21.94% of patients had experienced ≥ 10 to < 25 attacks. This appears to be generally in line with the global icatibant Outcome Survey, in which 8.2% of patients experienced 1–2 attacks, 23.5% experienced 3–6 attacks, 12.9% experienced 7–11 attacks, and 29.4% experienced 12–51 attacks annually [38]. With regard to the location of attacks in the 12 months prior to icatibant administration, 68.92% of patients experienced cutaneous attacks, 73.65% experienced abdominal attacks, and 27.03% experienced laryngeal attacks. This was consistent with a recent global chart review study of patients with suboptimally controlled HAE, in which 70.1% of patients reported abdominal attacks and 25.7% reported laryngeal attacks [39]. In the present study, there was a higher incidence of laryngeal edema among patients with HAE‐nC1INH than patients with HAE‐C1INH, which was also consistent with previous reports [32]. However, the designation “laryngeal attack” in the present study was based on reports by patients and physicians and thus may have included instances of swelling of different parts of the upper airway, such as the tongue and/or pharynx. Taken together, there appear to be no major differences in HAE attack characteristics between Japan and other countries.

During this study, a total of 377 HAE attacks were treated, including 234 episodes treated with icatibant alone and 48 episodes treated with icatibant plus other treatment(s). Most HAE attacks were treated by self‐administration of icatibant (237/377 attacks, 62.86%), consistent with self‐administration rates in the global Icatibant Outcome Survey (68.5%) [38] and in a telephone survey of patients with HAE in the United States (71.2%) [40]. These data indicate that many patients in real‐world settings self‐administer icatibant, allowing them to respond rapidly to acute attacks of HAE. In the Japanese phase 3 trial, the efficacy and tolerability of icatibant for acute attacks were demonstrated, regardless of self‐administration or administration by a healthcare professional [34].

Out of 234 HAE attacks in all patients treated with icatibant alone (*N* = 100), 228 episodes were treated with a single icatibant injection. By HAE type, 176 out of 182 episodes (96.70%) in patients with HAE‐C1INH and 18 out of 18 episodes (100.00%) in patients with HAE‐nC1INH were treated with a single injection; the remaining episodes occurred in patients with unknown HAE type. By contrast, attacks treated with other treatment(s) in addition to icatibant most commonly involved 2 treatment administrations per attack (up to 4), although it was not possible to determine whether the repeated treatment was icatibant or the concomitant medication(s). The high percentage of attacks treated with a single administration of icatibant is consistent with results from clinical trials and the international Icatibant Outcome Survey [35, 38].

Early symptom relief of acute HAE attacks was observed in patients treated with icatibant alone. Approximately 75% of patients treated with icatibant alone experienced symptom relief within 4 h, and approximately 60% achieved complete resolution of symptoms within 24 h. The median time to initial symptom relief was 60 min, and to complete resolution of symptoms was 710 min (11.8 h). These findings were broadly consistent with the Japanese phase 3 open‐label study results (0.98–1.04 h to initial symptom relief and 5.98 h to almost complete symptom relief [34]) and the global FAST clinical trials (0.8–1.5 h to initial symptom improvement, and 3.5–19.7 h to almost complete symptom relief [29, 32, 33]). The times to first symptom relief and to complete resolution of symptoms in the icatibant alone group were numerically shorter than the times in the group treated with multiple medications. However, this study lacks information on the drug sequence used in cases treated with multiple medications. Furthermore, there is no information about the relationship between treatment choice (icatibant alone vs. icatibant plus other medications) and the severity of each attack. In addition, the group treated with icatibant alone may have included a higher proportion of mild attacks.

In the present study, a higher proportion of patients with HAE‐C1INH initiated on‐demand treatment within 1 h of attack onset than previously reported [41]. In our cohort, approximately 25% of attacks were treated within 30 min and approximately 70% were treated within 4 h. In contrast, the initiation of treatment in patients with HAE‐nC1INH was generally delayed: only about 10% of patients initiated treatment within 30 min, < 30% within 1 h, and < 40% within 4 h. This suggests there may be differences in treatment initiation timing between patients with HAE‐C1INH and patients with HAE‐nC1INH when attacks occur. Earlier treatment may be beneficial, as previous evidence suggests that attack severity may increase with longer time to initiation of on‐demand treatment [41].

The proportion of patients with HAE‐nC1INH in the present study (14.19%) was similar to a previous Japanese study [23] but relatively high compared with previous reports in non‐Japanese countries [42]. However, recent estimates of the proportion of patients with HAE‐nC1INH in non‐Japanese countries have been higher than previously thought; for example, 27.6% of patients with HAE in a Brazilian registry [43] and 24% of patients at Angioedema Centers of Reference and Excellence worldwide (range: 2% [Australia/the Netherlands] to 44% [Austria]) were diagnosed with HAE‐nC1INH [44]. It is important to note that classification of patients into HAE types in the present study was based on CRF entries and was not determined independently. Moreover, because HAE‐nC1INH can be challenging to treat, patients with HAE‐nC1INH may have been more likely to receive icatibant and thus be included in this study, particularly as icatibant is indicated in Japan for all patients with HAE. Although patients with HAE‐nC1INH may respond to HAE therapies, including icatibant, treatment responses are often less robust than those observed in patients with HAE‐C1INH [16]. The evidence base for icatibant in subpopulations such as HAE‐nC1INH and HAE of unknown origin remains limited to case reports, case series, and small cohort studies [16, 45, 46].

Information about on‐demand treatment for pregnant and lactating patients with HAE is limited. Plasma‐derived C1‐INH is generally recommended as first‐line therapy [18, 26, 47, 48], but some patients continue to use icatibant during their pregnancy and lactation. In this study, maternal exposure to icatibant during pregnancy and/or breastfeeding was observed, and no adverse events were reported. These findings are consistent with published reports of safe and successful use of icatibant during pregnancy [49, 50, 51, 52, 53]. Although more experience is needed, no unfavorable infant health outcomes were identified in this study among patients exposed to icatibant during pregnancy and/or lactation.

Limitations of the study include the relatively small sample size and the limited observation period for HAE attacks and icatibant use. In particular, the numbers of patients who had either very severe attacks or who received inpatient treatment were small. In addition, information about HAE attacks relied on patients' recall. In Japanese post‐marketing surveillance, data collection is limited to CRFs completed by treating physicians, and source data verification of the recorded information cannot be performed. Because icatibant can be self‐administered, it is unclear whether all ADRs were reported to physicians and reflected in the CRF. Similarly, time to symptom relief or resolution was unknown for many patients, most likely reflecting an absence of information available to physicians completing the CRFs and reducing the data available for effectiveness analyses. The nature of post‐marketing data collection also affected the HAE subtype classifications and genetic mutation status: an “unknown” classification was allowed for both, as it was anticipated that some patients, such as those referred from other institutions, would not have a clearly documented HAE subtype or genetic testing recorded in their clinical information. Because the “unknown” classification was based on information available in routine clinical practice, the classification should be interpreted with caution. For this reason, HAE subtype‐stratified safety and effectiveness analyses were restricted to patients whose subtype (HAE‐C1INH or HAE‐nC1INH) could be confirmed. Effectiveness analyses were also restricted to patients for whom the use of icatibant was considered to be on‐label, so pediatric patients and patients without a confirmed HAE diagnosis were excluded from these analyses. Finally, because significance tests were not prespecified in this study, statistical comparisons among HAE types were not performed.

In conclusion, this post‐marketing surveillance study of icatibant adds to the real‐world evidence on the clinical characteristics and treatment experiences of Japanese patients with HAE, including patients with HAE‐nC1INH and HAE attack characteristics (location and severity). No serious ADRs were observed, no new safety concerns were identified, and icatibant was associated with early symptom relief in acute HAE attacks.

## Funding

This study was funded by Takeda Pharmaceutical Company Limited, manufacturer of icatibant. Medical writing assistance was provided by Koa Webster, PhD, CMPP and Rebecca Lew, PhD, CMPP of ProScribe KK, part of Envision Ignite, an Envision Medical Communications agency, a part of Envision Pharma Group, and was funded by Takeda. ProScribe's services complied with international guidelines for Good Publication Practice. Takeda Pharmaceutical Company Limited was involved in the study design, data collection, data analysis, and preparation of the manuscript.

## Conflicts of Interest

K.H. and M.H. are employees of Takeda Pharmaceutical Company Limited. M.H. is a shareholder of Takeda Pharmaceutical Company Limited.

## Supporting information


**Table S1:** List of concomitant medications and therapies (Safety Analysis Set).

## Data Availability

The datasets, including the redacted study protocol, redacted statistical analysis plan, and individual participants data supporting the results reported in this article, will be made available within 3 months from initial request, to researchers who provide a methodologically sound proposal upon reasonable request. The data will be provided after its de‐identification, in compliance with applicable privacy laws, data protection and requirements for consent and anonymization.
