# Supplementary material for: Real‐World Safety and Effectiveness of Icatibant in Patients With Hereditary Angioedema (HAE): Post‐Marketing Surveillance in Japan
Source: J Dermatol. 2026 Aug 21;53(11):1599–614. doi: 10.1111/1346-8138.70447 (PMC13737455; doi:10.1111/1346-8138.70447)
Supplement: Supplementary file 1 [file JDE-53-1599-s001.docx]

SUPPLEMENTARY MATERIAL

Real-World Safety and Effectiveness of Icatibant in Patients With Hereditary Angioedema (HAE): Post-Marketing Surveillance in Japan

Kumiko HOSONO, Miyako HOSHINO

Japan Medical Office, Takeda Pharmaceutical Company Limited, Tokyo, Japan

AUTHOR FOR CORRESPONDENCE

Kumiko HOSONO

Japan Medical Office

Takeda Pharmaceutical Company Limited

1-1, Nihonbashi-Honcho 2-chome

Chuo-ku, Tokyo, 103-8668

Japan

Email: kumiko.hosono@takeda.com

LIST OF TABLES

Supplementary Table 1	List of Concomitant Medications and Therapies (Safety Analysis Set)

**SUPPLEMENTARY TABLE 1** List of concomitant medications and therapies (Safety Analysis Set).

| **Nonproprietary name of medication** | **Drug code** | ***N* (%)^†,‡^** |
| --- | --- | --- |
| Tranexamic acid | 3327002 | 65 (57.52) |
| Plasma-derived C1-INH | 6343426 | 54 (47.79) |
| Danazol | 2499007 | 7 (6.19) |
| Famotidine | 2325003 | 4 (3.54) |
| Fexofenadine hydrochloride | 4490023 | 4 (3.54) |
| Investigational product | SHP99 | 4 (3.54) |
| Amlodipine besilate | 2171022 | 3 (2.65) |
| Warfarin potassium | 3332001 | 3 (2.65) |
| Metformin hydrochloride | 3962002 | 3 (2.65) |
| Bepotastine besilate | 4490022 | 3 (2.65) |
| Montelukast sodium | 4490026 | 3 (2.65) |
| Bilastine | 4490033 | 3 (2.65) |
| Sodium valproate | 1139004 | 2 (1.77) |
| Acetaminophen | 1141007 | 2 (1.77) |
| Loxoprofen sodium hydrate | 1149019 | 2 (1.77) |
| Scopolamine butylbromide | 1242002 | 2 (1.77) |
| Mometasone furoate hydrate | 1329710 | 2 (1.77) |
| Omalizumab (genetical recombination) | 2290400 | 2 (1.77) |
| Rebamipide | 2329021 | 2 (1.77) |
| Esomeprazole magnesium hydrate | 2329029 | 2 (1.77) |
| Vonoprazan fumarate | 2329030 | 2 (1.77) |
| Magnesium oxide | 2344002 | 2 (1.77) |
| Alfacalcidol | 3112001 | 2 (1.77) |
| Febuxostat | 3949003 | 2 (1.77) |
| Sitagliptin phosphate hydrate | 3969010 | 2 (1.77) |
| Vildagliptin | 3969011 | 2 (1.77) |
| **Concomitant Therapies** |  | |
| **Therapy** | ***N* (%)^§^** | |
| Artificial dialysis | 1 (33.33) | |
| Tracheostomy | 1 (33.33) | |
| Hemodialysis maintenance | 1 (33.33) | |

^†^Percentage of *N* = 113 patients with concomitant medications.

^‡^Only medications used by ≥2 patients are listed individually. Medication types used by only 1 patient each (0.88%) were alendronate sodium hydrate, ambroxol hydrochloride, amoxicillin hydrate, ascorbic acid, benidipine hydrochloride, betamethasone/d-chlorpheniramine malate, betamethasone sodium phosphate, bifidobacterium drug product (5), bixalomer, blonanserin, bucillamine, cabergoline, calcium l-aspartate hydrate, carvedilol, chlorpheniramine maleate, cilostazol, clarithromycin, clonazepam, clobazam, dabigatran etexilate methanesulfone, dapagliflozin propylene glycolate hydrate, desloratadine, dienogest, domperidone, dupilumab (genetical recombination), edoxaban tosilate hydrate, epinastine hydrochloride, epoetin beta pegol (genetical recombination), escitalopram oxalate, estazolam, eszopiclone, ethyl icosapentate, evocalcet, extract from inflamed cutaneous tissue of rabbits inoculated with vaccinia virus, ezetimibe, famotidine, fluticasone propionate/formoterol fumarate, gentamicin sulfate, hydrocortisone, ipragliflozin l-proline, kakkonto kagenkyukusinji, kambakutaisoto, lamotrigine, levothyroxine sodium hydrate, lithium carbonate, loxoprofen sodium hydrate calcitriol, maintenance solution (3), menatetrenone, metoclopramide, miglitol, morphine hydrochloride hydrate, naphazoline nitrate, olopatadine hydrochloride, omega-3-acid ethyl esters, parnaparin sodium, pentazocine, pitavastatin calcium, precipitated calcium carbonate, prednisolone, prednisolone valerate acetate, pregabalin, rikkunshito, rivaroxaban, rosuvastatin calcium, rupatadine fumarate, shin'iseihaito, sodium cromoglicate, sodium gualenate hydrate, sodium rabeprazole, sucroferric oxyhydroxide, suvorexant, teprenone, tranexamic acid (with different code to that in table), trazodone hydrochloride, triazolam, ursodeoxycholic acid, vilanterol trifenatate/fluticasone furoate, and yokukansan.

^§^Percentage of *N* = 3 patients with concomitant therapies.

Abbreviation: C1-INH, C1-inhibitor.
